# Development of a postoperative visual function rehabilitation compliance assessment scale for children with congenital cataract: a reliability and validity study

**DOI:** 10.1186/s40001-024-01922-4

**Published:** 2024-06-12

**Authors:** Yanan Zhang, Wanhua Xie, Daoman Xiang

**Affiliations:** 1 Ophthalmology Department, Guangzhou Women and Children’s Medical Center, Guangzhou Medical University, Guangdong Provincial Clinical Research Center for Child Health, Guangzhou, 510623 China; 2 Outpatient Department, Guangzhou Women and Children’s Medical Center, Guangzhou Medical University, Guangdong Provincial Clinical Research Center for Child Health, Guangzhou, 510623 China

**Keywords:** Congenital cataract, Visual function rehabilitation, Compliance, Scale development, Reliability, Validity, Interactive models of health behaviors

## Abstract

**Objective:**

To develop a comprehensive compliance assessment scale for postoperative visual function rehabilitation in children with congenital cataracts and to assess its reliability and validity.

**Method:**

Drawing on the Interactive Model of Health Behavior, we conducted a literature review and semi-structured interviews to create a pool of 36 items. The items underwent rigorous evaluation through the Delphi method, face validity checks, and item analysis, leading to a reduction to 18 items. To assess the scale's reliability and validity, we collected data from 225 parents of children with congenital cataracts. We employed SPSS version 25.0 for data analysis and evaluated construct validity using exploratory factor analysis, content validity, internal consistency reliability, and test–retest reliability.

**Results:**

The compliance scale for postoperative visual function rehabilitation in children with congenital cataracts comprises 5 dimensions and 18 items. Exploratory factor analysis extracted 5 common factors, with a cumulative variance contribution rate of 68.178%. Item-level content validity index ranged from 0.730 to 1.000, and the content validity index of the scale was 0.963. The total Cronbach's alpha coefficient, split-half reliability, and test–retest reliability of the scale were 0.855, 0.778, and 0.859, respectively.

**Conclusions:**

The compliance assessment scale for postoperative visual function rehabilitation in children with congenital cataracts demonstrates acceptable reliability and validity. It serves as a valuable reference for developing standardized nursing programs for these children in clinical practice.

**Supplementary Information:**

The online version contains supplementary material available at 10.1186/s40001-024-01922-4.

## Introduction

Congenital cataract, characterized by lens opacification in early infancy, is relatively prevalent in China, with an incidence ranging from 0.5 to 5.6‰. Notably, Asia bears the highest burden of congenital cataract [15]. Congenital cataract is mainly treated by surgery to remove the lens [8]. Nevertheless, the extent of postoperative visual recovery hinges on a protracted and methodical course of visual function rehabilitation (VFR) [14, 20, 25]. VFR, following congenital cataract surgery, encompasses refractive correction, patching therapy, and amblyopia training therapy. The level of compliance with VFR emerges as a critical determinant of its effectiveness [5, 16]. High compliance may compensate for suboptimal postoperative visual recovery due to treatment delays, while low compliance is directly linked to the development of amblyopia [1, 16]. Hence, it is of paramount importance to precisely evaluate postoperative VFR compliance in children and promptly implement standardized nursing interventions. However, existing assessment tools concerning visual function compliance predominantly comprise self-compiled questionnaires [5, 7, 17]. Cao [2] conducted a survey on the compliance of infants wearing glasses after congenital cataract surgery using a questionnaire containing 11 questions (e.g., “Is your infant’s spectacles frame soft?”). The survey investigated the compliance level and influencing factors for infants with congenital cataracts after surgery. However, the questionnaire lacked a reliability and validity test, which often lack rigor, comprehensiveness, and the consideration of external factors. Furthermore, they are generally more suitable for assessing compliance in children with amblyopia and those undergoing patch-up therapy, thus lacking specificity in evaluating VFR compliance in children with congenital cataract [9, 23]. Felius [9] evaluated the Amblyopia Treatment Index questionnaire (ATI) for assessing the impact of amblyopia on the psychological characteristics and treatment burden of children and their parents. The treatment compliance subscale in the ATI consists of five items primarily related to postoperative eye drops. It includes both a child version (e.g., “Using treatment bothers me”) and a parent version (e.g., “I have difficulty giving my child eye drops”). The overall Cronbach’s α coefficient for the scale was 0.88, and for the treatment compliance subscale, it was 0.84, indicating good reliability and validity. The scale is suitable for psychological status testing of children with amblyopia and their parents, involving the compliance of children with visual rehabilitation. However, there are shortcomings in assessing compliance with visual rehabilitation training, as the scale only addresses the use of eye drops and does not consider the long-term recovery process after surgery. It is also unclear whether the scale is applicable to children under 3 years of age. Additionally, these existing tools do not encompass evaluations of medical staff, environmental factors, parental involvement, and other facets of compliance, rendering them inadequate for accurately assessing postoperative VFR compliance in children with congenital cataract. Therefore, there is a lack of a scientifically reliable scale for assessing the compliance of infants with congenital cataracts throughout the entire process of visual rehabilitation after surgery.

Cox’s Health Behavior Interaction Model (IMCHB) is a nursing practice model that systematically guides nursing assessment, interventions, and outcomes. It can be used to systematically guide nursing research and promote the development and application of nursing interventions. The model elucidates the impact of clients' background factors, intrinsic motivation, cognitive assessment, emotional responses, and their interactions with healthcare professionals on health-related behaviors [6]. It underscores the interactions and mutual contributions of clients, families, and healthcare professionals in shaping and promoting health behaviors.

IMCHB is particularly well-suited for promoting health behaviors during the transition period and within a home setting, particularly in the context of chronic diseases [4, 12]. Since the rehabilitation of visual function in children with congenital cataract after surgery primarily occurs at home and demands long-term adherence to the rehabilitation regimen, this approach is highly relevant. IMCHB guided the development of the postoperative VFR compliance assessment tool for children with congenital cataract. Consequently, it ensures comprehensive coverage of compliance by children, caregivers, and medical staff, making the scale applicable to the nursing care of congenital cataract. The adherence of children to their treatment regimen is influenced by their unique characteristics and their interactions with healthcare educators [6]. The uniqueness of children is shaped by various factors, including background elements, intrinsic motivation, cognitive evaluation, and emotional responses. Taking into account the specific features of postoperative VFR in children with congenital cataract, this study initially formulated the postoperative Visual Function Rehabilitation Compliance Scale, encompassing five dimensions: ① Background Factors: Comprising family and social influences and environmental resources. ② Intrinsic Motivation: Encouraging patients to recognize their abilities and autonomy, along with self-rewards. ③ Cognitive Evaluation: Assessing patients' perceptions of their health status and how it influences their health-related behaviors. ④ Emotional Response: Addressing the emotional experiences that affect health behaviors and can either hinder or promote cognitive activities. ⑤ Interaction: Involving medical staff support, which encompasses health-related information, professional skills, emotional assistance, and shared decision-making tailored to the unique characteristics of children [4, 19, 24].

In light of these considerations, this study developed the postoperative VFR compliance assessment scale for children with congenital cataract based on the IMCHB. The reliability and validity of the scale were tested, providing an effective tool for assessing the postoperative VFR compliance of children with congenital cataract.

## Methods

**Fig. 1:**
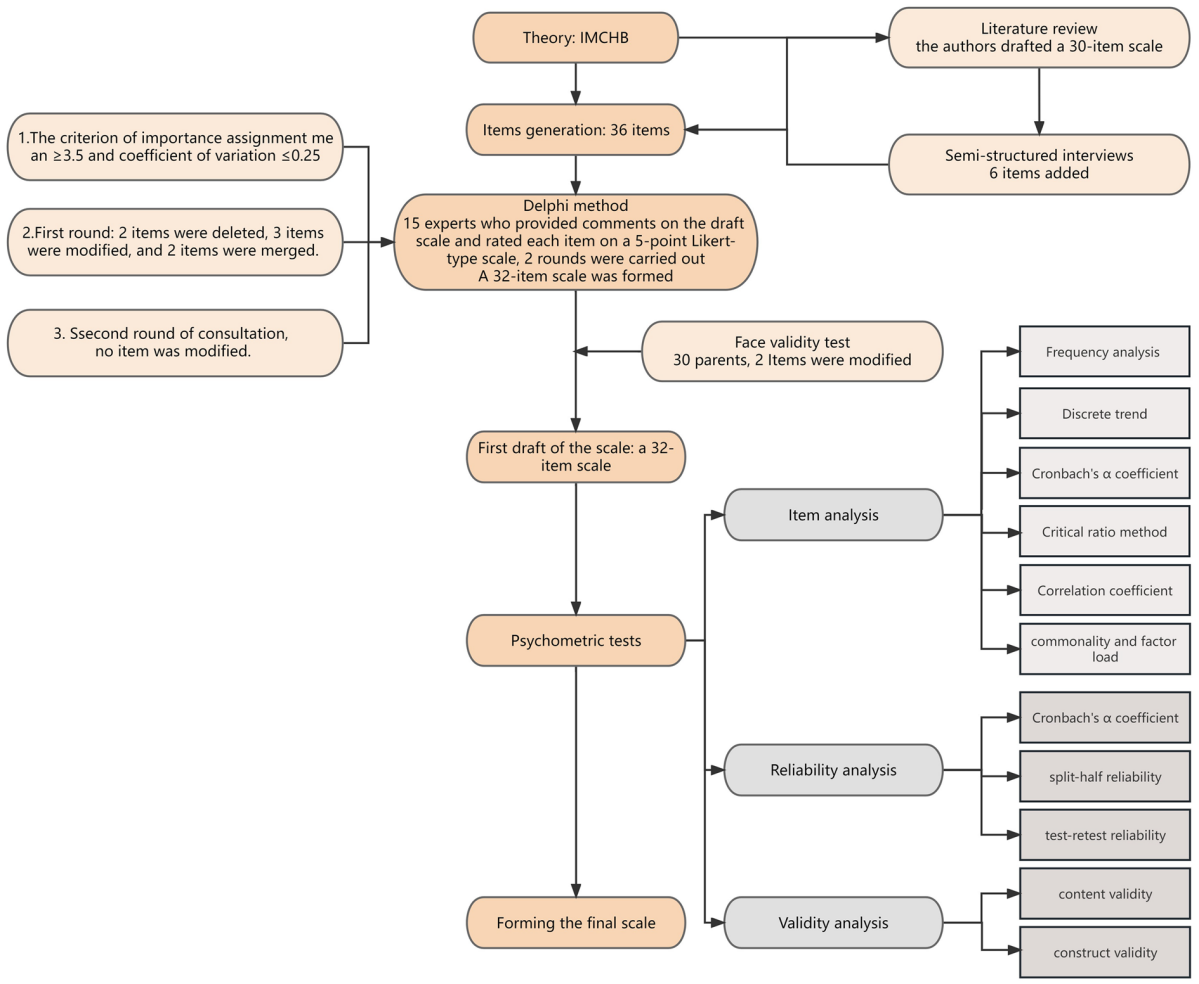
Flowchart

This study comprised two distinct phases: scale development and the assessment of its reliability and validity. The scale’s development involved a comprehensive approach, incorporating literature reviews, semi-structured interviews, the Delphi method, and face validity assessments. To gauge the validity of the scale, both construct and content validity measures were employed. Meanwhile, the scale's reliability was assessed through internal consistency and test–retest reliability evaluations. For a detailed overview of the process, refer to Fig. 1. This study has been approved by the Ethics Committee (2022209A01).

### Development of the scale

#### Literature review

“Congenital cataract/pediatric cataract/infant/children/pediatrics/visual function rehabilitation/refractive correction/occlusion treatment/amblyopia training/questionnaire/measurement/assessment/scale development/scale customization/reliability/validity” were used as search terms. CNKI, Wanfang, SinoMed, PubMed, Embase, and Web of Science were searched from the establishment of the database to February 2022. Inclusion Criteria: (a) Study Population: Infants with congenital cataracts and their caregivers. (b) Published literature addressing postoperative visual function recovery compliance in infants with congenital cataracts, including both qualitative and quantitative studies. Exclusion Criteria: (a) Literature available only in abstract form and inaccessible in full. (b) Repetitive publications or literature with incomplete data. (c) Literature not published in Chinese or English. Related literature was read and collected, and a scale pool containing 30 items was initially developed based on IMCHB.

#### Semi-structured interview

Using purposive sampling, caregivers of children with congenital cataract from both outpatient and inpatient departments were selected as research participants. They were chosen based on the maximum difference method, considering factors such as age, gender, disease type, duration of VFR for the children, as well as the gender, age, education background, and family residence of the caregivers. The sample size was determined by the principle of data saturation, and there were no refusals or dropouts during the study. Thirteen parents of children with congenital cataract treated in the ophthalmology clinic and inpatient department of a specialized children's hospital in Guangzhou from February to April 2022 were selected for semi-structured interviews. Inclusion criteria for children with congenital cataract were: ① the presence of lens opacities at birth or within the first year after birth; ② children aged ≤ 15 years in need of VFR after surgery. Exclusion criteria for children with congenital cataract included: ① severe infections and malignant tumors; ② retinopathy of prematurity, endophthalmitis, outer exudative retinitis, retinoblastoma, and other causes of VFR.

For caregivers of children with congenital cataract, inclusion criteria were: ① primary caregivers responsible for more than half of the prescribed VFR duration by the doctor; ② those who provided informed consent and voluntarily participated in the study; ③ caregivers with normal comprehension and language expression abilities. The exclusion criterion for caregivers was dropping out from the study.

The interview data was meticulously coded using Nvivo 11.0 software following the phenomenological research method. Two researchers employed the Colaizzi 7-step method for data analysis, sorting, summarization, and comparative analysis. Interview Outline: ① How compliant is your child with visual rehabilitation, and what challenges have you encountered? ② What factors do you believe influence your child's compliance? ③ What effective methods have you used to enhance children's compliance with eye patch treatment? ④ What kind of assistance do you hope to receive while aiding children in visual function rehabilitation?

The research team analyzed the interview data and incorporated six items derived from the interview content.

#### The Delphi method

The inclusion criteria for experts were as follows: ① Doctors should hold a master's degree or higher, and nurses should have at least a bachelor's degree; ② Hold an intermediate or higher professional title; ③ Be engaged in children's ophthalmology, nursing management, clinical nursing, psychology, or related fields, with over 10 years of work experience; ④ Have a genuine interest in this study. The accuracy of the correspondence results was found to be positively correlated with the number of correspondents. However, too many experts would increase the complexity of data analysis and processing. Therefore, a selection of 15 experts was made.

The consultation questionnaire consisted of three parts: consultation instructions, the questionnaire itself, and basic information about the experts. The consultation instructions encompassed the research background, purpose, significance, the definition of related concepts, an introduction to the Delphi expert consultation, and instructions for completion. The questionnaire text listed the items and dimensions of the postoperative visual function rehabilitation compliance scale for children with congenital cataract. A Likert 5-scale method was used to assess the items and dimensions, with ratings ranging from 1 to 5, representing “Not important” to “Very important.” Simultaneously, content validity was evaluated. A Likert 3-scale method was employed to evaluate the applicability of each item and dimension, with ratings from 1 to 3, indicating “Not applicable” to “Applicable.” Experts were also asked to provide explanations for their choice to apply or not apply after modification. Basic information about the experts included their education background, professional title, research field, and years of work experience. Experts self-assessed their familiarity with this research question, considering event experience, theoretical analysis, references to domestic and foreign literature, and an intuitive sense.

Between April and June 2022, 15 experts were selected to evaluate the items of the scale. Researchers provided detailed information about the study's purpose, significance, theoretical basis, and research methods to the experts. Communication with experts was conducted through telephone, WeChat, or email to ensure their full understanding and engagement with the study. After two rounds of consultation, expert opinions tended to converge, marking the conclusion of the consultation process.

Based on the criterion of an importance assignment mean of ≥ 3.5 and a coefficient of variation of ≤ 0.25 [3], the scale's items were either supplemented, deleted, or modified. Following the expert consultation results, the items were revised and supplemented, resulting in the first draft of the postoperative visual function rehabilitation compliance scale for children with congenital cataract. In the first round of consultation, two items were deleted, three items were modified, and two items were merged. In the second round of consultation, no items were modified. The revised scale consisted of 32 items.

#### Face validity test

Convenience sampling was employed to recruit 30 parents of children with congenital cataract who received treatment at a children's hospital's Ophthalmology clinic in Guangzhou for a preliminary assessment of the scale items' readability and feasibility in June 2022. Thirty questionnaires were distributed and collected on-site. In preliminary studies, the sample size typically ranges from 15 to 30 participants, aiming to represent the target population [26]. The inclusion and exclusion criteria aligned with those of the semi-structured interview.

### Scale reliability and validity

#### Study participants

Parents of children with congenital cataract who visited both the outpatient and inpatient departments of a children’s hospital in Guangzhou between July 2022 and July 2023 were chosen as survey participants using a convenient sampling method. The inclusion and exclusion criteria were consistent with those used in the semi-structured interviews. As for sample size calculation, it’s recommended that the sample size for factor analysis should be 5–10 times the number of items in the scale, and it should ideally exceed 200 cases [18, 26]. The initial scale comprised 32 items, and with a 10% inefficiency margin, the required sample size was 225 cases.

#### Data collection

The general information questionnaire was developed by the research team, drawing from relevant literature. It encompassed details such as gender, age, cataract type, complications, and the age and gender of the parents.

The initial version of the Postoperative Visual Rehabilitation Compliance scale for Children with Congenital Cataract featured five dimensions and a total of 32 items. Each item was rated using a Likert 5-point scale, with “Completely inconsistent” scored as 1 point, “Less consistent” as 2 points, “Generally consistent” as 3 points, “More consistent” as 4 points, and “Completely consistent” as 5 points. Notably, three items, namely “My child is unwilling to accept visual function rehabilitation,” “My child engages in non-serious behaviors like stealing and slacking during visual function rehabilitation,” and “I find it challenging to facilitate my child's visual function rehabilitation,” were scored in reverse.

The researchers provided a uniform explanation of the study’s purpose and significance to the parents. After obtaining their consent, the questionnaires were completed on-site with one-on-one guidance from the researchers, ensuring the data's completeness by addressing any missing items as they arose.

#### Data analysis

Frequency analysis method, discrete trend method, critical ratio method, correlation coefficient method, Cronbach's α coefficient, commonality and factor load were used to test the discrimination of each item. Item deletion criteria: ① Frequency analysis method: Responses were focused on specific choices (over 80%) or an option was not answered at all to consider removal. ② Discrete trend method: items with standard deviation < 0.75 should be deleted [13]. ③ Critical ratio method: the scale scores were sorted from small to large, and the corresponding values of 27% and 73% were used as the upper limit value and the lower limit value for dividing the low group and the high group, respectively, to compare whether there was any difference in the scores of each item between the low group and the high group. If there was no difference in the scores of the two groups, the discrimination of the item was poor, and it was considered for deletion. ④ Correlation coefficient method: if the correlation coefficient with the total score of the scale was not statistically significant or less than 0.3, it was considered to delete the items [3]. ⑤ Cronbach’s α coefficient: after removing an item, Cronbach's α coefficient increased greatly, so consider deleting it,⑥commonality and factor load: the items with factor loading value ≥ 0.4 were retained.

Item-level content validity index (I-CVI) of postoperative visual function rehabilitation compliance scale for children with congenital cataract and scale-level content validity index (S-CVI). If I-CVI ≥ 0.70 and S-CVI ≥ 0.80, the content validity of the scale was good [3]. Exploratory Factor Analysis (EFA) was used to test the construct validity of the scale. In the exploratory factor analysis, the Kaiser–Meyer–Olkin (KMO) index and Bartlett’s sphericity test were applied to confirm that the data were appropriate for factor analysis. The KMO index ranged from 0 to 1, with the closer to 1 indicating a stronger correlation between the variables the more suitable for factor analysis; Principal component analysis (PCA) and maximum variation method were used for orthogonal axis rotation. The common factors with eigenvalues > 1 and cumulative variance contribution rate > 40% were selected, and the items with factor loading value ≥ 0.5 were retained.

Cronbach’s α coefficient and split-half reliability were used to evaluate the internal consistency of the total score and the scores of each dimension of the scale. In the fourth week after the formal investigation, 30 subjects were selected for repeated measurement to evaluate the test–retest reliability of the scale.

EpiData software was used to input and organize data. SPSS25.0 statistical software was used for data processing. Measurement data were expressed as (mean ± standard deviation), enumeration data were expressed as numbers (percentage), and authority coefficient, effective questionnaire recovery rate, Kendall harmony coefficient and coefficient of variation were expressed as authority coefficient of experts, enthusiasm, coordination degree of opinions and moderation degree of opinions, respectively.* p* < 0.05 was considered statistically significant.

## Results

### Delphi method and face validity test results

In this study, a total of 15 experts were selected. Their age was 36 to 58 (48.06 ± 8.07) years, and their working years in related fields were 13 to 38 (25.47 ± 8.18) years. The panel included 8 ophthalmic medical experts and 7 ophthalmic nursing experts, with 8 holding senior titles, 5 holding associate senior titles, and 2 holding intermediate titles. These experts came from various regions in China, including Shanghai, Guangdong, Yunnan, Hebei, Henan, Fujian, and Guangxi.

The response rate for both rounds of questionnaires was 100%. During the two rounds of expert consultation, the response rate for each question was consistently high, ranging from 93.33 to 100%, and 100%. Furthermore, 46.67% (7 out of 15) of experts provided constructive revision suggestions in the first round, and 6.67% (1 out of 15) did so in the second round, resulting in a total of 12 expert opinions received. The expert judgment coefficient (Ca) was 0.82, the expert familiarity index (Cs) was 0.83, and the expert authority coefficient (Cr) was 0.84. The coordination coefficient of expert opinions was 0.205 (*p* < 0.001). The coefficient of variation (CV) for the experts’ scores on the importance of each item ranged from 0.125 to 0.248. The importance scores of each item ranged from 4.00 to 4.75, with full score ratios ranging from 0.27 to 0.87.

According to the importance of items, the scale was scored and revised by experts. After the discussion of the research team, the experts proposed to ①Add “careless behavior” and revise it to “careless behavior such as slacking and peeking.” ② Delete “I will try to communicate with the child and carry out visual function rehabilitation” because most of the children are infants and have no cognitive ability. ③ Supplement I am very concerned about the child's future vision (and willing to work hard for it). ④ Combined the medical staff will regularly ask about my recovery, and the medical staff will regularly evaluate my recovery and give opinions. ⑤ Modify the statement “I can’t help my child to rehabilitate visual function” to “it is difficult for me to rehabilitate my child's visual function”. ⑥ Modify the statement “The attitudes and views of others around me will affect my enthusiasm for the rehabilitation of my child” to “The attitudes of other parents will affect my enthusiasm for the rehabilitation of my child”.

The time of 30 caregivers to complete the pre-survey scale was (13.46 ± 8.68) minutes, and 2 items were modified according to the feedback. “My child cannot accept visual function rehabilitation” was revised to “My child is not willing to undergo visual function rehabilitation”; “I think my family life environment is very stable and there is no significant change” was revised to “My child’s visual function rehabilitation has no effect on my family life”. Finally, a test version of the postoperative VFR compliance assessment scale for children with congenital cataract was formed with 32 items.

### General information of respondents

A total of 230 questionnaires were distributed and 225 valid questionnaires were collected, with an effective recovery rate of 97.83%. There were 149 males (66.20%) and 76 females (33.80%), with an average age of (4.32 ± 2.72) years. 78 (34.7%) were unilateral and 147 (65.30%) were bilateral. 178 (79.10%) had no complications and 47 (20.90%) had complications. There were 68 fathers (30.20%), 150 mothers (66.70%), and 7 others (3.10%), with an average age of (33.13 ± 5.644) years. There were 112(49.78%) families living in city and 113 (50.22%) families living in countryside. There were 116 (51.56%) families with average monthly income less than 5,000, 80 (35.56%) families with average monthly income between 5000 and 800,018 (8.00%) families with average monthly income between 8000 and 20,000, and 11(4.89%) families with average monthly income more than 20,000 (Table 1).

**Table 1:** Demographic characteristics of the participants

Category	*N* = 225
Age (years)	4.32 ± 2.72
Age of parent	33.13 ± 5.64
Type of CC (bilateral)	147 (65.30%)
Role (mother)	150 (66.70%)
(Father)	68 (32.20%)
(Others)	7 (3.10%)
Type of CC (bilateral)	147 (65.30%)
Complication (No)	178 (79.10%)
Residential location (City)	112 (49.78%)
(Countryside)	113 (50.22%)
Family average monthly income(< 5000RMB)	116 (51.56%)
(5000–8000RMB)	80 (35.56%)
(8000–20,000RMB)	18 (8.00%)
(> 20,000RMB)	11 (4.89%)

### Results of item analysis

Frequency analysis method showed that the response rate of each item of the 32 items in the questionnaire was 100%, and no item had a frequency of more than 80% on an option, and no item had a frequency of 0 on an option. Discrete trend method showed that the standard deviation of 3 items was less than 0.75. Critical ratio method showed that 2 items with no difference between the two groups (*p* > 0.05). Correlation coefficient method showed that the correlation coefficient between the scores of 7 items and the total score of the scale was less than 0.3. Cronbach’s alpha coefficient method showed that no items met the deletion criteria, commonality and factor load method showed that 2 items < 0.4. According to the comprehensive screening strategy, 14 items were finally removed.

### Validity analysis

#### Content validity

I-CVI of the Postoperative Visual Rehabilitation Compliance scale for children with congenital cataract ranged from 0.730 to 1.000. The S-CVI was 0.963.

#### Construct validity

**Fig. 2:**
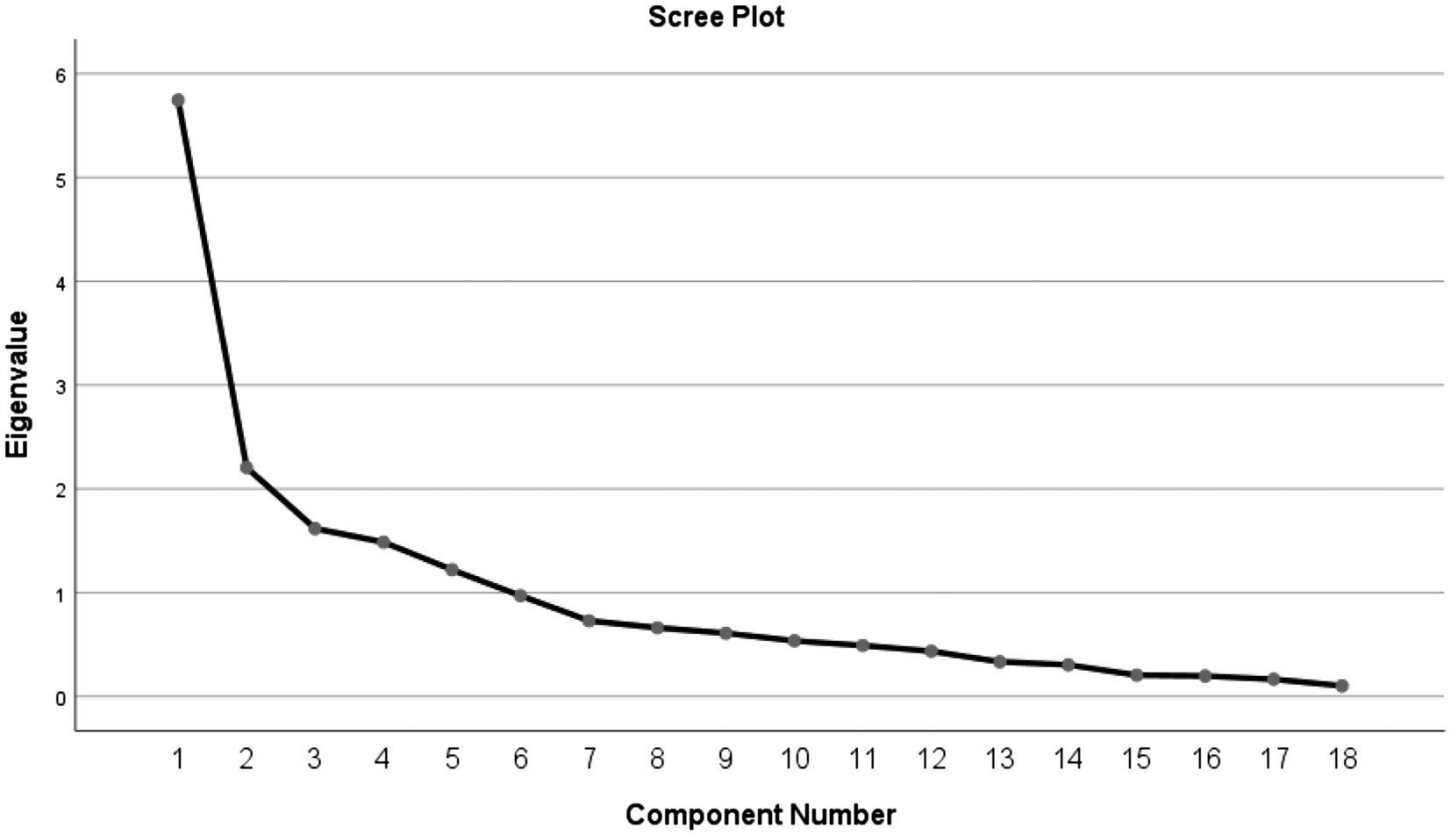
Scree plot of the scale (18 items)

EFA was used to test the construct validity of the scale, and Bartlett's spherical test *x*^*2*^ value was 2070.843 (*p* < 0.001), KMO index was 0.734 > 0.7, indicating that it was suitable for factor analysis. A total of 5 factors with eigenvalues > 1 were extracted by principal component analysis and maximum variance rotation, and the cumulative variance contribution rate was 68.178% (Table 2). They were named as background factors, intrinsic motivation, cognitive evaluation, emotional response, and interaction. The load matrix of exploratory factor analysis is shown in Table 3. Figure 2 is the Scree plot of the scale (18 items).

**Table 2:** Results of principal component analysis of test scale items

Total variance explained									
Component	Initial eigenvalues			Extraction sums of squared loadings			Rotation sums of squared loadings		
	Total	% of variance	Cumulative %	Total	% of variance	Cumulative %	Total	% of variance	Cumulative %
1	5.745	31.919	31.919	5.745	31.919	31.919	3.163	17.573	17.573
2	2.205	12.249	44.168	2.205	12.249	44.168	2.820	15.665	33.238
3	1.616	8.981	53.148	1.616	8.981	53.148	2.425	13.472	46.710
4	1.485	8.252	61.400	1.485	8.252	61.400	2.029	11.270	57.980
5	1.220	6.778	68.178	1.220	6.778	68.178	1.836	10.199	68.178
6	0.970	5.387	73.565						
7	0.728	4.045	77.610						
8	0.661	3.670	81.279						
9	0.608	3.380	84.659						
10	0.535	2.975	87.634						
11	0.490	2.723	90.357						
12	0.435	2.416	92.773						
13	0.333	1.848	94.622						
14	0.303	1.683	96.304						
15	0.204	1.133	97.437						
16	0.196	1.089	98.526						
17	0.165	0.914	99.440						
18	0.101	0.560	100.000						

Extraction method: principal component analysis

**Table 3:** Exploratory factor analysis results of compliance scale for postoperative visual function rehabilitation in children with congenital cataract (*n* = 225)

Item	Interaction	Intrinsic motivation	Emotional reaction	Cognitive appraisal	Background factor
1.When I did not achieve the expected recovery effect, the medical staff would give me timely guidance	**0.840**	0.137	0.147	0.254	0.009
2. Medical staff will ask regularly to assess the child's recovery and give advice	**0.809**	− 0.053	0.039	− 0.022	0.251
3. I can get timely guidance when I seek help from medical staff for problems in my recovery	**0.783**	0.224	0.176	0.141	0.019
4. Medical staff will guide and help me with visual rehabilitation methods	**0.655**	0.28	0.229	0.237	0.027
5. When my child has blurred vision (redness and swelling, wound bleeding and other conditions) I will seek medical attention in time	0.127	**0.765**	− 0.132	0.015	0.085
6. I believe that postoperative visual function rehabilitation can promote visual recovery	0.220	**0.701**	0.318	0.202	0.066
7. I care very much about my child's vision in the future and am willing to work hard for it	0.208	**0.680**	0.409	0.018	− 0.021
8. I will patiently guide children to cooperate when they resist	-0.195	**0.641**	0.143	0.137	0.320
9. My family members and I have the same view on how to carry out the child's rehabilitation	0.255	**0.502**	0.377	0.047	− 0.112
10. In the process of recovery, I can get encouragement and supervision from my family members	0.211	0.350	**0.788**	− 0.03	0.066
11. When I encounter problems in visual rehabilitation for my child, my family members will assist me	0.120	0.359	**0.769**	0.026	-0.034
12. I have plenty of time for my child's visual rehabilitation	0.075	− 0.190	**0.749**	0.307	0.168
13. Since the beginning of rehabilitation, my child has been rehabilitating according to the regulations, and sometimes the compliance is poor	0.161	− 0.069	0.048	**0.841**	0.088
14. My child is capable of visual rehabilitation as long as prescribed	0.109	0.179	0.033	**0.832**	− 0.022
15. I can help children complete visual rehabilitation	0.227	0.305	0.284	**0.559**	0.228
16. I will choose video, painting and other ways to guide children to recover	− 0.179	0.219	0.089	0.049	**0.865**
17. I understand the specific methods and processes of children's visual function rehabilitation	0.421	0.046	0.050	0.043	**0.670**
18. There are patients around me who encourage and communicate with each other about their feelings of visual rehabilitation	0.432	− 0.040	− 0.025	0.122	**0.594**

Some values are bolded because the loadings for those items in the respective dimensions are greater than 0.5. These items were retained and included in the final scale

### Reliability analysis

The Cronbach’s α coefficient of the scale was 0.855, and the Cronbach's α coefficient of each dimension was 0.850, 0.725, 0.746, 0.720, 0.658. The total split-half reliability of the scale was 0.778, and the split-half reliability of each dimension was 0.787, 0.779, 0.787, 0.646, 0.644. The total test–retest reliability of the scale was 0.859, and the test–retest reliability of each dimension was 0.898, 0.822, 0.740, 0.847, 0.754 (Table 4).

**Table 4:** Reliability of the scale

	Cronbach's α coefficient	Split-half reliability	Test-retest reliability
Interaction	0.850	0.787	0.898
Intrinsic motivation	0.725	0.779	0.822
Cognitive evaluation	0.746	0.787	0.740
Emotional response	0.720	0.646	0.847
Background factors	0.658	0.644	0.754
Scale	0.855	0.778	0.859

## Discussion

### The postoperative visual function rehabilitation compliance scale for children with congenital cataract, constructed in this study, is scientifically developed

It was formulated based on the theoretical framework of IMCHB. The first draft of the scale was meticulously prepared through a comprehensive literature review and semi-structured interviews. This process was guided by a scientific and standardized theoretical foundation. Furthermore, semi-structured interviews were conducted, employing a purposive sampling method to ensure maximum differentiation among the samples and reduce potential biases. The scale items were refined through the rigorous Delphi method. All 15 experts involved in the process were professionals in relevant fields, holding intermediate or higher professional titles. Eight of them possessed master's degrees or higher qualifications, showcasing their strong academic research capabilities. The positive coefficient for the two rounds of expert consultation was 100%, demonstrating the high enthusiasm of these experts in participating in the study.

The experts’ authority coefficients were impressive, with a value of 0.84 and Cr ≥ 0.8, indicating their high level of expertise [21]. Kendall’s harmony coefficient was calculated to be 0.205 (*p* < 0.001), and the coefficient of variation ranged from 0.125 to 0.248, all exceeding 0.25, which signifies a high level of agreement among the experts and good coordination. The importance scores for each item ranged from 4.00 to 4.75, all surpassing 3.5. The full score ratio ranged from 0.27 to 0.87, all exceeding 0.20, underscoring the high level of consensus among experts regarding the importance of each item.

EFA identified a total of 5 common factors with characteristic values exceeding 1. The final scale encompasses 5 dimensions: background factors, intrinsic motivation, cognitive evaluation, emotional response, and interaction. These dimensions are consistent with the theoretical framework of IMCHB, and the attribution of other dimensions and items closely aligns with the theoretical hypothesis. The interaction between health educators and patients constitutes the core component of IMCHB, including affective support, provision of health information, decisional control, and professional or technical competencies [6]. Items 1 to 4 are designed to depict healthcare professionals assessing the child's condition and providing professional information and guidance for postoperative visual function rehabilitation, aligning with the interactive component of IMCHB. Items 5 to 9 describe the parents' self-perceived reasons for adhering to visual function rehabilitation and unexpected situations, reflecting the patient's sense of self-efficacy and autonomy, consistent with the intrinsic motivation of health behavior theories. Items 10 to 12 illustrate the emotional responses of parents during the child’s rehabilitation process, considering the surrounding environment such as companions and family, correlating with the emotional response aspect of IMCHB. Cognitive appraisal, consisting of knowledge, attitudes, beliefs about health, treatment, and health behaviors in IMCHB, aligns with Items 13 to 15, which describe the parents' cognitive assessment of the child's compliance with rehabilitation. The background variables in IMCHB include demographic characteristics, social influences, medical history, and environmental resources. Items 16 to 18 address social influences (family, peer, and cultural influences) and environmental resources (availability of informational, people, financial, and geographic resources to facilitate health behavior) pertaining to the child's background, constituting part of the background factors in IMCHB. However, the lack of medical history, demographic characteristics, or the involvement of too many factors may contribute to the dimension's Cronbach’s alpha coefficient being below 0.7. Each item exhibited a loading value on the common factor greater than 0.4, signifying the stability of the scale's structure [3]. The cumulative variance contribution rate reached 68.178%, implying that the scale effectively captures the comprehensive aspects of postoperative compliance in children with congenital cataract. Furthermore, the I-CVI exceeded 0.70, and the S-CVI was no less than 0.80, demonstrating the scale's robust content validity [18]. Specifically, in this study, the I-CVI and S-CVI were recorded at 0.730 to 1.000 and 0.963, respectively, underscoring the high content validity of the postoperative visual function rehabilitation compliance scale for children with congenital cataract.

The scale demonstrated strong internal consistency, with the Cronbach's α coefficient exceeding 0.8. Additionally, each dimension exhibited a Cronbach's α coefficient surpassing 0.6, and the split-half reliability exceeded 0.7, collectively indicating the scale's robust internal consistency [10]. However, both split-half reliability and Cronbach's α coefficient can be influenced by sample size, and given the relatively small sample size in this study, the results of split-half reliability may be less stable, and Cronbach's alpha coefficient may be overestimated. The fifth dimension, which pertains to background factors, displayed a Cronbach’s α coefficient below 0.7. This dimension comprises items such as “I will choose video, painting and other ways to guide children to recover”, “I understand the specific methods and processes of children's visual function rehabilitation”, “There are patients around me who encourage and communicate with each other about their feelings of visual rehabilitation”. This lower coefficient may be attributed to a plethora of background factors. Furthermore, the scale exhibited strong test–retest reliability, with a coefficient exceeding 0.7, signifying its good stability over time and external consistency [3]. In this study, considering that compliance is unlikely to be affected in the short term, the time interval for test–retest reliability was set at 4 weeks, relatively longer compared to other studies. However, over time, participants may forget details of their previous responses, affecting their consistency and potentially leading to a decrease in the study's reliability.

In summary, the development process of the postoperative visual rehabilitation compliance scale for children with congenital cataract is both scientific and standardized, and it demonstrates good reliability and validity.

### The postoperative visual function rehabilitation compliance scale for children with congenital cataract constructed in this study is practical

The postoperative visual function rehabilitation compliance scale developed in this study is practical and crucial for evaluating compliance among children with congenital cataract, thus forming the foundation for standardizing postoperative visual function rehabilitation and enhancing visual recovery in these patients.

Having precise compliance assessment tools is essential for healthcare professionals to implement targeted nursing interventions and offer health guidance. Presently, there is no standardized assessment scale for compliance in children with congenital cataract. Many rely on self-designed questionnaires, which often lack rigor, comprehensiveness, and the consideration of external factors. Typically, these questionnaires primarily focus on the perceptions and attitudes of both children and their parents [11, 22]. In contrast, the questionnaire developed in this study encompasses five dimensions. It provides a more comprehensive view of the cognitive, motivational, and emotional aspects, as well as the interactions between doctors, nurses, and patients during the VFR process for children with congenital cataract. This approach aligns with the IMCHB model.

This scale is well-suited for dynamic assessments of children's compliance at different phases of home rehabilitation, enabling healthcare providers to identify the underlying factors influencing children's health behaviors and compliance with minimal resource expenditure. The study primarily assesses the sensitivity of the scale over time through test–retest reliability. The participants included in this study were not differentiated based on the duration of visual function rehabilitation, aiming to encompass various stages of rehabilitation. However, this study only retested the reliability after 4 weeks, which is relatively short compared to the duration of postoperative visual function rehabilitation. Therefore, further applied research is needed to explore the time sensitivity of the scale. Moreover, the questionnaire in this study is concise, consisting of only 18 items. This brevity ensures quick completion and facilitates its practical application in clinical settings. During the investigation, participants may be influenced by the researcher's identity, leading them to provide responses that align with social expectations rather than their genuine individual experiences. This tendency can impact the reliability of research results. Therefore, mitigating the influence of social expectations as much as possible poses a significant challenge in the study. Additionally, given the extended duration of the visual rehabilitation process following congenital cataract surgery, minimizing participant attrition during follow-up is also a substantial challenge.

The main limitations of this study are as follows: the samples were only from a children's hospital in Guangzhou, and the source of samples was limited; No confirmatory factor analysis was performed. It is suggested that the sample size should be increased in the future, and the relevant visual function rehabilitation compliance scale should be selected as the calibration questionnaire to further verify the structural validity and calibration association validity of this scale.

## Conclusion

The visual function rehabilitation compliance scale for children with congenital cataract developed in this study is scientific and practical and can be used as a measurement tool for medical staff to continuously and dynamically evaluate the visual function rehabilitation compliance of children with congenital cataract after surgery.

### Supplementary Information

Supplementary Material 1.

Supplementary Material 2.

## Data Availability

The data used in this study were collected by field investigation, and all authors agree to provide relevant data as far as possible without affecting the privacy of patients.
