# Supplementary material for: Development of a postoperative visual function rehabilitation compliance assessment scale for children with congenital cataract: a reliability and validity study
Source: Eur J Med Res. 2024 Jun 12;29:324. doi: 10.1186/s40001-024-01922-4 (PMC11167793; doi:10.1186/s40001-024-01922-4)
Supplement: Supplementary file 1 — Supplementary Material 1. [file 40001_2024_1922_MOESM1_ESM.docx]

**Postoperative Visual Function Rehabilitation Compliance assessment Scale for Children with Congenital Cataract**

| Item | Completely inconsistent | Less consistent | Generally consistent | More consistent | Completely consistent |
| --- | --- | --- | --- | --- | --- |
| **Interaction** |  |  |  |  |  |
| 1.When I did not achieve the expected recovery effect, the medical staff would give me timely guidance |  |  |  |  |  |
| 2. Medical staff will ask regularly to assess the child's recovery and give advice |  |  |  |  |  |
| 3. I can get timely guidance when I seek help from medical staff for problems in my recovery |  |  |  |  |  |
| 4. Medical staff will guide and help me with visual rehabilitation methods |  |  |  |  |  |
| **Intrinsic motivation** |  |  |  |  |  |
| 1. When my child has blurred vision (redness and swelling, wound bleeding and other conditions) I will seek medical attention in time |  |  |  |  |  |
| 2. I believe that postoperative visual function rehabilitation can promote visual recovery |  |  |  |  |  |
| 3. I care very much about my child's vision in the future and am willing to work hard for it |  |  |  |  |  |
| 4. I will patiently guide children to cooperate when they resist |  |  |  |  |  |
| 5. My family members and I have the same view on how to carry out the child's rehabilitation |  |  |  |  |  |
| **Emotional reaction** |  |  |  |  |  |
| 1. In the process of recovery, I can get encouragement and supervision from my family members |  |  |  |  |  |
| 2. When I encounter problems in visual rehabilitation for my child, my family members will assist me |  |  |  |  |  |
| 3. I have plenty of time for my child's visual rehabilitation |  |  |  |  |  |
| **Cognitive appraisal** |  |  |  |  |  |
| 1. Since the beginning of rehabilitation, my child has been rehabilitating according to the regulations, and sometimes the compliance is poor |  |  |  |  |  |
| 2. My child is capable of visual rehabilitation as long as prescribed |  |  |  |  |  |
| 3. I can help children complete visual rehabilitation |  |  |  |  |  |
|  |  |  |  |  |  |
|  |  |  |  |  |  |
| Item | Completely inconsistent | Less consistent | Generally consistent | More consistent | Completely consistent |
| **Background factor** |  |  |  |  |  |
| 1. I will choose video, painting and other ways to guide children to recover |  |  |  |  |  |
| 2. I understand the specific methods and processes of children's visual function rehabilitation |  |  |  |  |  |
| 3. There are patients around me who encourage and communicate with each other about their feelings of visual rehabilitation |  |  |  |  |  |
