# Supplementary material for: Development of a postoperative visual function rehabilitation compliance assessment scale for children with congenital cataract: a reliability and validity study
Source: Eur J Med Res. 2024 Jun 12;29:324. doi: 10.1186/s40001-024-01922-4 (PMC11167793; doi:10.1186/s40001-024-01922-4)
Supplement: Supplementary file 2 — Supplementary Material 2. [file 40001_2024_1922_MOESM2_ESM.docx]

Number of records after duplicates removed

(N=872)

Number of records excluded (N=811)

•Irrelevant based on title (n =766)

•Irrelevant based on abstract (n =44)

• Non-Chinese and English (n= 1)

Number of records screened (title and abstract) n=61

Number of articles excluded on reading full-text (N=33)

Content not relevant to compliance (n =28)

The research object is not consistent (n = 2)

Data cannot be separated (n = 1)

Unable to obtain original text (n = 1)

Number of full-text articles assessed for eligibility

(N=28)

Number of articles included

**(N=28)**

Number of articles assessed for quality (N=28)

The literature with poor quality was excluded quality rating C

(N=0)

1067 records **identified**

PubMed (n = 656), The Cochrane Library (n = 123), Web of Science (n = 87) ), Scopus (n=94, CNKI (n = 40), WanFang (n = 43), CBM (n =24)

Number of additional records identified through other sources (N=0)

**Identification**

**Screening**

**Eligibility**

**Included**

**Figure 1. PRISMA flow diagram of search and study selection process**
